# Review of Solutions for the Use of Phase Change Materials in Geopolymers

**DOI:** 10.3390/ma14206044

**Published:** 2021-10-13

**Authors:** Michał Łach, Kinga Pławecka, Agnieszka Bąk, Marcin Adamczyk, Patrycja Bazan, Barbara Kozub, Kinga Korniejenko, Wei-Ting Lin

**Affiliations:** 1 Chair of Materials Engineering, Faculty of Material Engineering and Physics, Krakow University of Technology, Jana Pawła II 37, 31-864 Krakow, Poland; michal.lach@pk.edu.pl (M.Ł.); agnieszka.bak@pk.edu.pl (A.B.); patrycja.bazan@pk.edu.pl (P.B.); barbara.kozub@pk.edu.pl (B.K.); kinga.korniejenko@pk.edu.pl (K.K.); 2 Chair of Building Materials Engineering, Faculty of Civil Engineering, Krakow University of Technology, 31-155 Krakow, Poland; marcin.adamczyk@pk.edu.pl; 3 Department of Civil Engineering, National Ilan University, No. 1, Sec. 1, Shennong Rd., Yilan City 260, Taiwan; wtlin@niu.edu.tw

**Keywords:** geopolymer, phase change materials, sustainable material technologies, building composites

## Abstract

The paper deals with the possibility of using Phase Change Materials (PCM) in concretes and geopolymer composites. The article presents the most important properties of PCM materials, their types, and their characteristics. A review of the latest research results related to their use in geopolymer materials is presented. The benefits of using PCM in building materials include the improvement of thermal comfort inside the building, and also the fact that the additive in the form of PCM reduces thermal gradients and unifies the temperature inside the concrete mix, which can reduce the risk of cracking. The paper also presents a critical analysis related to the feasibility of mass scale implementations of such composites. It was found that the use of PCM in sustainable construction is necessary and inevitable, and will bring a number of benefits, but it still requires large financial resources and time for more comprehensive research. Despite the fact that PCM materials have been known for many years, it is necessary to refine their form to very stable phases that can be used in general construction as well as to develop them in a cost-effective form. The selection of these materials should also be based on the knowledge of the matrix material.

## 1. Introduction

Energy is a key element in assessing the progress of society around the world in various aspects such as technological development, environmental protection, and economic progress. The continuous depletion of non-renewable energy resources leads to the major problem of global warming. This is prompting scientists to change their approach for better and cleaner use of energy. With a higher standard of living and technological growth, the electricity demand is increasing day by day, leading to excessive consumption of fossil fuels. Minimizing the amount of consumed energy can reduce environmental pollution and also have a positive impact on the energy market.

The production of the most important building material of the 20th century, Portland cement, is associated with significant environmental pollution and energy problems. It is produced at very high temperatures of the order of 1400–1500 °C. During production, significant amounts of carbon dioxide and highly toxic nitrogen oxides are emitted into the atmosphere. The building sector is responsible for huge energy consumption which is estimated to be around 40% worldwide—of which, residential buildings consume 27% of energy and contribute 17% of CO_2_ emissions to the environment [1]. This is due to rapid population growth and changing standards of lifestyle. Such high energy demand is reflected in significant environmental pollution and degradation. To avoid the negative effects of excessive energy consumption, sustainable building materials are increasingly being developed [2]. In recent years, sustainable technologies and materials for the construction sector have begun to develop very quickly in Poland and in the rest of the world.

In recent years, alkali-activated binders/geopolymer cement and concrete seem to be an alternative to conventional concrete. Alkali activated binders are produced by alkaline activation of aluminosilicate raw materials such as blast furnace slag (GGBFS), fly ash (FA), metakaolin (MK), etc. These binders can solve the problems of the construction industry and waste generation. Therefore, since calcium-rich raw materials, especially slags, are limited, more attention is paid to raw materials with a low calcium content, from which geopolymers are produced [3]. In 1978, Joseph Davidovitz first coined the term “geopolymer” [4]. The term geopolymer covers the class of modern, environmentally-friendly, inorganic, amorphous, synthetic aluminosilicates with specific composition and properties [5]. Geopolymers are three-dimensional aluminosilicate framework structures from amorphous to semi-crystalline, which are formed by a combination of [SiO_4_]^4−^ and [AlO_4_]^5−^ tetrahedra. The structure remains electrically neutral as a result of the replacement of aluminum by silicon in the tetrahedral layer with available alkalis, such as Na^+^ [6]. The mechanism of setting and hardening of geopolymer is not fully understood. The geopolymerization process can be carried out by dissolving raw materials containing aluminosilicate in alkaline solutions, which leads to the formation of aluminate and silicate monomers. Then they turn into oligomers and then into geopolymers [5]. Research on geopolymers is conducted mainly to replace conventional cement with them and, consequently, to use them on a large scale in construction. Concrete made from geopolymer cements has received considerable attention because of their low cost, good engineering properties as well as low energy consumption. Materials of this type are used in specific areas, where their unique properties, such as very high fire resistance, chemical corrosion resistance, high mechanical strength, and excellent durability, are important [3,7,8,9,10,11,12]. Geopolymers are characterized by better performance compared to concrete and, importantly, large amounts of by-products (fly ash, coal ash, and blast furnace slag) are used on production [13,14,15,16,17,18].

Despite the existence of building materials that are more environmentally friendly than conventional cement, there is still a need to improve the energy efficiency of buildings and reduce emissions of substances that are harmful to the earth’s atmosphere.

One of the solutions to reduce the negative impact on the environment is the application of phase change materials (PCM) [19]. Due to the declining supply of fossil fuels, in the last few years, phase change materials have attracted the interest of a wide range of researchers, organizations, and benefactors as they store thermal energy and release it when needed [20,21]. This is evidenced by research in various fields, such as the electronics industry [22], photovoltaic and solar systems [23,24], hot water systems [25].

**Figure 1:**
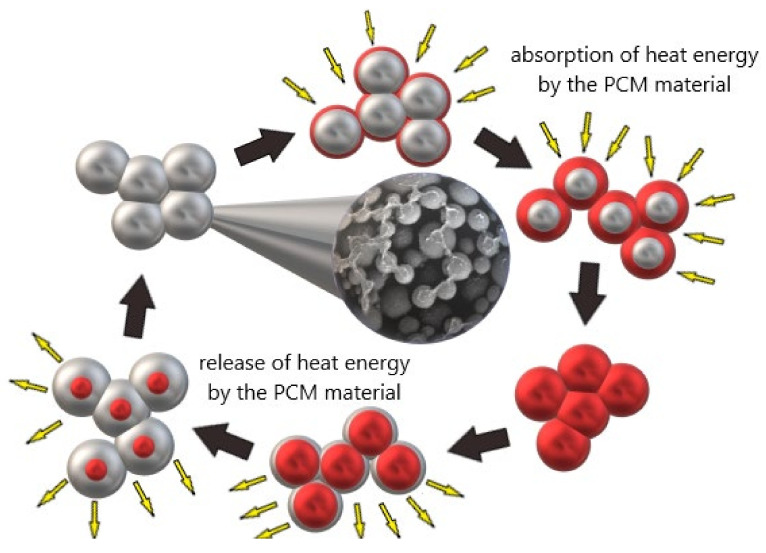
The mode of action of phase change materials.

Phase change materials (PCM) were known already in the 1970s (similarly to geopolymers), but their development is observed today. This is related to the possibility of obtaining a stable phase as well as the possibility of optimization of construction materials concerning the construction needs. These materials can change their physical state depending on the ambient temperature. The most common method is liquid-solid conversion. In the area of application of phase change materials in the construction industry, examples of such materials include organic materials (paraffin, fatty acids), inorganic materials, and eutectics. Most inorganic PCMs are characterized by large volume changes and potential subcooling, which has led to the wider use of organic PCMs in combination with concrete [26]. The most commonly used organic materials are in the form of microcapsules. Organic paraffin was considered as the best phase change material for concrete mortar due to its stability and inertness in an alkaline environment [27]. However, this material also has some limitations, such as low thermal conductivity, flammability, incompatibility with plastics, and large volume changes [28]. The choice of PCM material and the method of its incorporation is determined by e.g., the temperature range of phase transitions, the effective heat capacity, and the stability of the material during cyclic transitions. Phase change materials can have up to several times higher heat capacity than traditional construction materials. It is extremely important to use phase change materials in appropriate types of building materials. As research indicates, geopolymers may be a better option for these types of systems than traditional building materials [29]. The way PCM works in geopolymers is shown schematically in Figure 1.

The incorporation of microencapsulated phase change materials (MPCMs) in structural applications can improve the thermal energy storage capacity, thereby lowering the energy demand in buildings [30]. The addition of MPCM reduces the workability and mechanical strength of concrete. However, the type, quantity, and proportion of raw materials curing time, and temperature should be considered when formulating geopolymer concrete (GPC) [31].

Phase change materials have a high latent heat storage density, so they can absorb thermal energy when they transform from a solid to a liquid or release it when they transform to a solid state. Due to this property, PCM can function as a heating and cooling system in the building industry. During the day it absorbs excess thermal energy in a building component through a melting process, while at reduced temperatures (at night) it solidifies and releases thermal energy back into the environment.

PCM can be introduced into building components in three ways. The first method is to add it directly during the mixing process. Another method is to immerse the building structure in liquid PCM. The third method is the most advanced and popular, and consists of encapsulation of micro or macro PCM. It allows for better particle dispersion, reduces external volume changes, but also eliminates interaction between the phase change material and the starting material. Microencapsulation of phase change material is now an industrialized process, so it is very expensive and the product itself has been limited to only a few companies worldwide [32]. The addition of phase change materials to the inner layer of the partition will produce several beneficial phenomena not only related to thermal comfort inside the building [33,34,35] but also, among others, that the addition in the form of PCM reduces thermal gradients and unifies the temperature inside the concrete mix, which may reduce the risk of cracking. Phase change materials can also be added to interior finishes such as gypsum wallboard [36,37]. The addition of such materials results in a reduction of costs of heating or cooling buildings, and an increase in energy efficiency in rooms as well as increased comfort for users.

In the following part of the paper, the latest developments related to the application of PCMs in geopolymers materials, their characteristics, and method of incorporation into the geopolymer matrix are presented. This paper also presents the comments related to the real possibility of implementing such solutions on a mass scale.

## 2. Characteristics and Classification of Phase Change Materials

A material with a phase change is a compound or a group of compounds capable of absorbing and releasing large amounts of energy depending on the phase change they are currently in. The basic properties characterizing PCM are primarily the ability to heat accumulation and thermal conductivity of the substance. In addition, the behavior of the substance in superheated and subcooled conditions. These materials are characterized by a certain range and value of temperature at which the phase transformation occurs [38,39]. Phase change materials are also known as materials with the ability to accumulate so-called “latent” heat. The transfer of thermal energy takes place when a material changes from one phase to another, i.e., from a liquid to a solid or from a solid to a liquid state. Initially, these materials behave like conventional heat accumulators such as water—they increase in temperature as heat is supplied. However, PCMs absorb and release heat at an almost constant temperature. They can absorb 5 to 14 times more heat than standard materials [40]. To realize the full potential of phase change materials, they must meet some requirements from thermal, physical, chemical, and kinetic aspects. In addition, an important aspect of using a particular type of material is the economic aspect and the availability of materials [41,42].

Considering thermal issues, these materials should have high latent heat per unit volume. This aspect is extremely important because of the potential size of the heat accumulator. With high latent heat, the size of the heat storage can be minimized. The high thermal conductivity of such a material significantly facilitates the heat loading and unloading process. Small volume changes during phase transitions and low vapor pressure at working temperatures are other basic characteristics that PCMs should meet from the physical point of view. The basic chemical requirements for PCM are non-toxicity, non-flammability, and low probability of an explosion. During selections of phase change materials, it must be remembered not to subcool them, as this significantly impedes the dissipation of stored heat. Subcooling of the PCM by as little as 5–10 °C can cause complete blocking of the ability to transfer stored heat [40,41,42,43].

**Figure 2:**
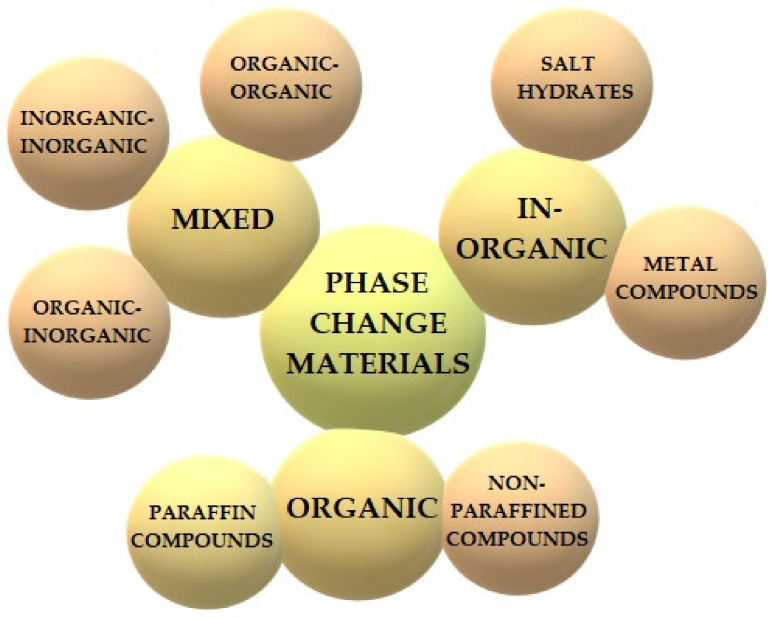
Classifications of phase change materials.

The basic criterion for the distinction of phase change materials is the structure and chemical composition. There are three basic classifications of phase change materials: organic, non-organic materials, and mixed (eutectic). Figure 2 shows the main types of phase change materials.

The use of a particular type of PCM has its advantages and disadvantages compared to other types. Based on the literature sources, several advantages and disadvantages for organic and inorganic PCMs were summarized. Starting from organic PCMs, their greatest advantages are compatibility with conventional common materials and high synthesis temperature [24]. In addition, they have self-nucleating properties and are safe and non-toxic. The disadvantages of organic PCMs include flammability and relatively low volumetric capacity to store latent heat [44]. They are also characterized by low thermal conductivity. In the case of non-organic materials, their greatest advantage is their low cost and high availability. Unlike organic materials, they are characterized by high latent heat storage capacity and high thermal conductivity. Like organic materials, they have a high synthesis temperature. Considering the most important disadvantages of inorganic phase change materials are their corrosion and frequent volume change in some mixtures. In addition, in the case of this type of PCM there is a high risk of subcooling of the substance [21].

Currently, dozens of large companies are involved in the production of phase change materials as well as finished products. The most well-known include, among others: Rubitherm, Doerken, BASF (Berlin, Germany), PCM Products (Peterborough, UK), PureTemp LLC (Minneapolis, USA), Climator (Skovde, Sweden), Cristopia (Vence, France), Mitsubishi Chemical (Kurashiki, Japan), TEAP Energy (Melbourne, Australia), PCM (Suzhou, China), PlusPolymer (Haryna, India) [45]. 

**Figure 3:**
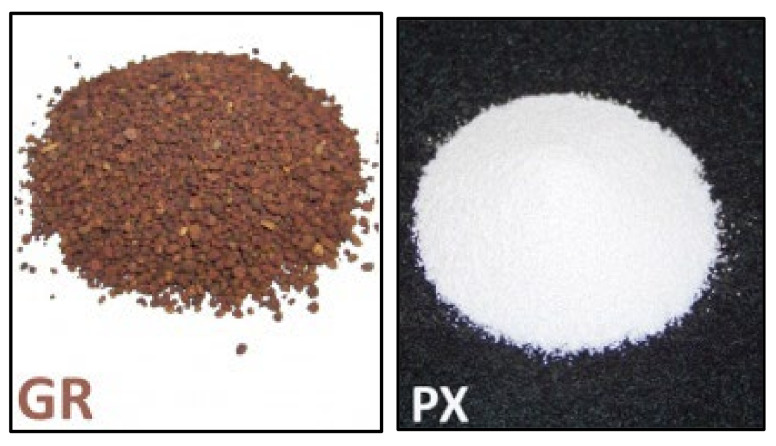
PCM granules manufactured by RUBITHERM [46].

A very interesting solution is the PCM materials introduced by e.g., RUBITHERM in the form of dry powder (PX) and granulates (GR) in an inorganic matrix. These should not be confused with microencapsulated PCM. Their appearance is shown in Figure 3. The organic part in PX material is about 60% while in GR variety it is 30%. The particle size of the PX inorganic carrier matrix is 200 µm. According to the manufacturer, PX materials are also more economical compared to microencapsulated PCM, but it should be taken into account that microencapsulated PCM cannot be replaced. The diameter of GR granules is about 1–3 mm. GR is used in the food and beverage industry and as a filler for accumulation plates [46].

Various manufacturers also offer PCM materials in other forms: encapsulated in plastic beads, stainless steel, steel tubes, pouches, ceramic materials. There are more and more solutions on the market, but many of them are designed and manufactured for food manufacturers and packaging manufacturers. There is also great development in the field of energy storage. However, there is still a lot of room for improvement when it comes to building materials. It should be remembered that this is a huge sector that can contribute to a significant increase in demand for MSM and consequently reduce the environmental impact on people. But this requires continuous improvement of these materials and adapting them to changing technologies.

## 3. Methods of Incorporation of PCM into Geopolymers and Concrete

One of the most significant problems in creating geopolymer composites with PCMs is how to place the PCM materials in the matrix. This is also true for conventional concrete composites. Using the wrong form may result in the little effect of the PCM addition, or the entire PCM material may be destroyed or removed after some time.

There are 4 main methods of incorporation of PCM into geopolymer:

- Microencapsulation (PCMs are enclosed in spherical shells),
- Shape stabilized PCMs (Molten PCM is directly absorbed into powdered porous structure like graphite powder, silica fume, etc. having higher thermal conductivity),
- Porous aggregate inclusions (Molten PCM is absorbed or impregnated into porous light weight aggregates having large surface area and aggregate size such as expanded perlite, expanded clay, pumice, etc.),
- Macroencapsulation (PCMs are incorporated into building components such as roofs or walls).

The use of stabilized forms of PCM (i.e., encapsulated or impregnated PCM) in porous support materials, such as expanded graphite or expanded perlite, in geopolymer mortar, can result in better mechanical and thermal properties compared to geopolymer containing neat PCM alone [2]. Besides, usage of PCM in free form—not encapsulated or encapsulated—can lead to several problems related to the spillage of PCM content outside the materials.

**Figure 4:**
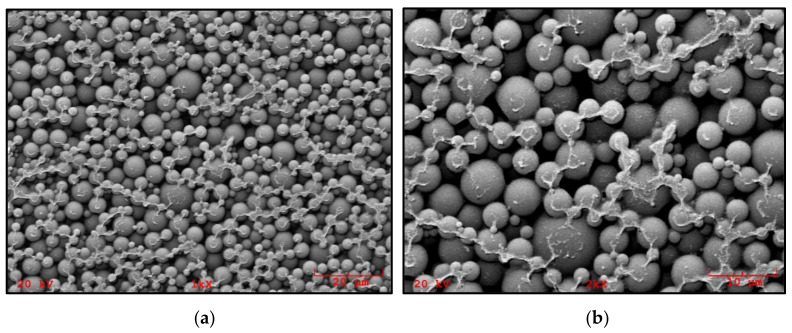
Example morphology of PCM microcapsules (manufacturer Microcaps; Slovenia): (**a**) 1000× magnification, (**b**) 2000× magnification (visible connection—lines are remnants of the emulsion in which PCM were immersed).

PCM or paraffin can be introduced into concrete by mixing with the other ingredients. Paraffin can be introduced into the concrete between the pores, aggregate, and cement slurry. However, after many cycles of temperature changes, paraffin leaks to the surface of the concrete. A solution to this problem has been found and paraffin is encapsulated or stored in encapsulated spheres to prevent leakage. The encapsulation is effective in terms of solving the leakage problem. However, the process is quite expensive, and because of the smooth surface, in some cases, it forms a weak bond with the cement slurry. Hermitization can be divided into macroencapsulation and microencapsulation. Macroencapsulation has quite a few limitations due to the solidification of PCM around the edges during heat recovery from the liquid phase, which prevents efficient heat transport. Microencapsulation results have shown improved thermal storage of walls with encapsulated PCM compared to traditional concrete without PCM due to better thermal inertia and low internal temperature [31]. An example of the appearance of PCM microcapsules for the construction industry is shown in Figure 4 (SEM image).

**Figure 5:**
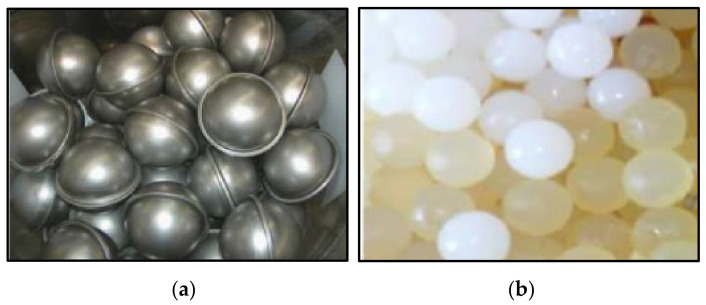
Organic PCM capsules: (**a**) in stainless steel matrix; (**b**) in HDPE matrix [45].

If due to cost or other reasons, it is not possible to use microencapsulated PCMs, other solutions can be used but it is not a simple matter. As our experience shows, the introduction of PCM in liquid form into a geopolymer matrix is not easy. Our research presents the degradation of gelatin and cellulose capsules, which were used to encapsulate organic PCM to place it together with the capsule in geopolymers. The attempts made were unsuccessful due to the degradation of the capsules (made of both gelatin and cellulose) after contact with the paraffin material. The only solution seems to be microencapsulation or encapsulation at the manufacturing stage and the purchase of ready encapsulated materials from the manufacturer. A possible solution can be also encapsulating organic liquids/paraffin in stainless steel (Figure 5a) and high-density polyethylene (HDPE) (Figure 5b) materials, as it was done for BallICE materials [45] (solutions used by TEAP Energy [47] and PCM Products [48]).

**Figure 6:**
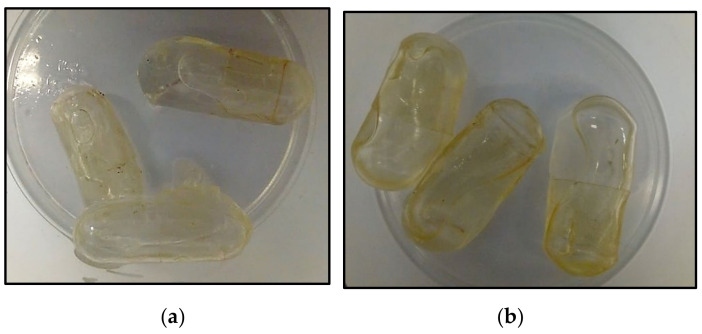
Degradation of capsules (shells) by contact with PCM (paraffin): (**a**) cellulose capsules; (**b**) gelatin capsules.

As mentioned before, the use of liquid paraffin as a PCM for residential applications, i.e., with a transformation temperature range of up to max. 28–30 °C is very problematic. Their main disadvantage is low thermal conductivity, a decrease in the rate of accumulation and release of heat during melting and crystallization (a large surface area is required), flammability, and density change. Moreover, changes in density during heating/cooling to the melting/solidification temperature and solid/liquid phase transition cause a large volume change. Figure 6 shows the effect of a phase change material based on paraffin on (a) cellulose capsules, (b) gelatin capsules.

**Figure 7:**
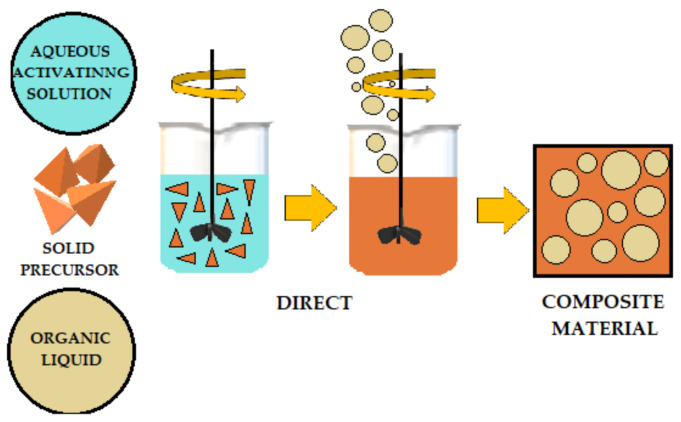
Schematic of direct addition of organic liquids to OPC (ordinary Portland cement) and AAM (alkali-activated) materials.

**Figure 8:**
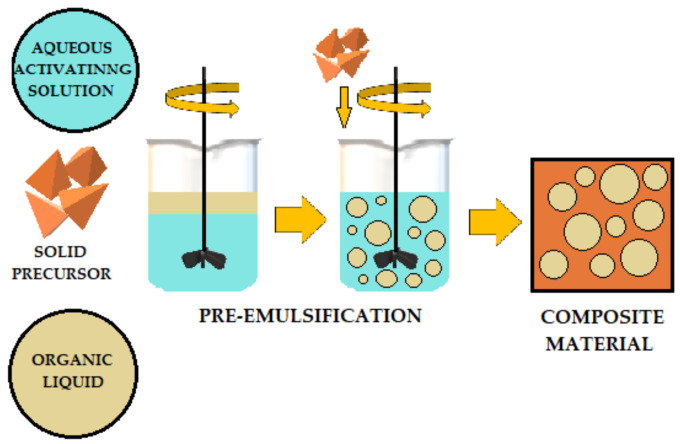
Preemulsification schematic of organic liquids added to OPC (ordinary Portland cement) and AAM (alkali-activated) materials.

**Figure 9:**
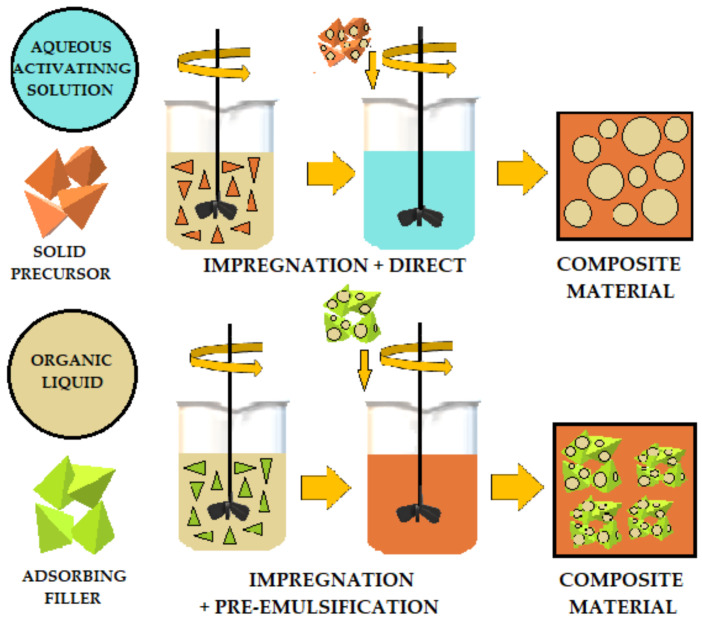
Combined method of adding organic liquids added to OPC (ordinary Portland cement) and AAM (alkali activated) materials.

The literature describes various methods for adding organic liquids to cement-based or geopolymer-based materials. Figure 7, Figure 8 and Figure 9 schematically illustrates three ways to introduce organic liquids into ordinary Portland cement (OPC) or geopolymers (GP). Table 1 compares these methods and describes their advantages and disadvantages [49].

## 4. PCM in Geopolymers—Effect on Insulation Properties

The use of PCM materials in geopolymers were widely described in [50,51,52], among others. This idea came about the same time that PCMs were added and studied in conventional concretes. Since it is, however, a different type of matrix, often with a very high pH, the issue of PCM addition to geopolymers should be considered separately from concretes based on Portland cement, even though the properties of such composites and their application may be similar.

Selected results of studies on geopolymers with PCM are presented below, mainly with respect to the influence of PCM on thermal properties of geopolymer composites. Only selected results are presented, and the focus is on presenting different forms in which PCM were introduced e.g., powder, encapsulated in graphite, encapsulated in polyurethane foams, microcapsules, encapsulated PCM, etc. Table 2 summarizes the most important information.

**Figure 10:**
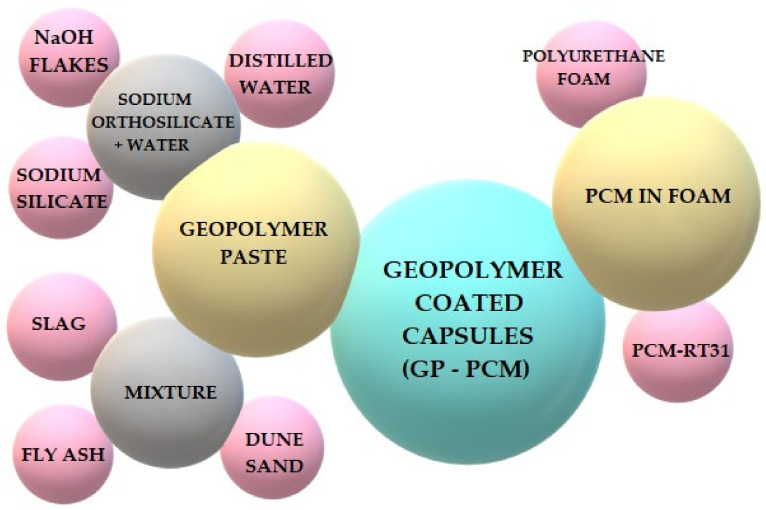
Raw materials and manufacturing process for PCM capsules.

Hassan et al., conducted research on phase change materials in geopolymers. The work was carried out in the United Arab Emirates with the cooperation of New Zealand. The phase change material was purchased from the German company Rubitherm with the trademark RT-31. Polyurethane foam was used as the matrix material to hold the PCM, which was coated with a geopolymer paste to produce form-stable PCM capsules (GP-F-PCM). The raw materials that were used to develop the GP-F-PCM capsules and the capsule procedure are shown in Figure 10 [53].

GPC cubes with dimensions of 50 mm × 50 mm × 50 mm were cast. Foam capsules and GP-F-PCM were added to the GPC cubes in proportions of 25%, 50%, and 75%. The thermal and structural properties were compared with the reference sample made of neat geopolymer. The results revealed that the addition of foam to GPC caused increase in the back surface temperature of the cubes, namely for 75% foam addition to GPC the back surface temperature increased by 5.8 °C compared to the reference cube for which the temperature was equal to 57 °C. On the other hand, the addition of GP-F-PCM capsules to the GPC allowed a decrease in the back surface temperature of the cube. The decrease in this temperature was greater the greater the addition of GP-F-PCM. The lowest temperature of 44.4 °C was obtained for the sample with the addition of 75% GP-F-PCM capsules. The study showed that polyurethane foam or GP-F-PCM could reduce the heat transfer in building spaces, while the direct integration of foam with GPC was harmful. The introduction of 75% foam very slightly increased the strength of the GPC cube (+3.6%) compared to the reference material. However, the strength decreased significantly when PCM capsules were introduced. After 28 days, the compressive strength went from 65.2 MPa for the reference sample to 9.9 MPa for the 75% GP-F-PCM composites. The results showed that the introduction of GP-F-PCM capsules provided the highest thermal performance compared to foam [53].

Another study was conducted in Arizona by Shadnia et al., Geopolymer mortar with an incorporated phase change material was tested. The phase change material in powder form (MPCM) was used. MPCMs are two-component particles containing a core material (PCM) and an outer shell or capsule wall. The capsule wall was made of inert, stable plastic. The PCM in the capsule melted at 28 °C, but the polymer coating was designed not to melt under processing and use conditions. From the experimental study, it was observed that the unit weight of the geopolymer mortar decreased when PCM was added because it had a low unit weight. The lower unit weight that was obtained after the introduction of PCM will result in a reduction in the weight of the geopolymer mortar wall and consequently the total weight of the building, which is very beneficial for the construction of lightweight buildings. The addition of PCM also led to a slight decrease in the compressive strength of the geopolymer mortar. However, this decrease was quite low and the compressive strength of the mortar was high enough for building applications [54].

SEM studies by the authors showed that the number of broken particles on the failure surface of geopolymer mortar samples increased with the introduction of more and more PCMs. The SEM images indicated that PCMs had a good bonding with the geopolymer binder, which explains why the compressive strength of geopolymer mortar with up to 20% PCM content was still high enough. The specific heat of the geopolymer mortar increased after the introduction of PCM. Thus, the introduced PCM could effectively reduce the heat transport through the geopolymer mortar. Thermal studies proved that PCM-doped geopolymer mortar can be used as a building wall to effectively increase the thermal inertia of buildings and reduce the energy demand during cooling and heating [54].

Afolabi et al., tested red mud geopolymer composite with phase change material. The research was conducted in Malta in collaboration with Nigeria. The geopolymer composite wall was made of red mud (Trussing Minning, Trussing, Australia), quartz sand (Tronoh in Perak, Malaysia), and PCM capsules encapsulated in expanded graphite (Avantis Company Laboratorium Berhad, Ipoh, Perak, Malaysia). Studies have shown that the mechanical strength and thermal properties were comparable to other wall materials, but due to the usage of waste materials, the cost of manufacturing was much lower. The phase change material was chemically and thermally stable within the expanded graphite. The sodium hydroxide formed a capsule-shaped barrier therefore no paraffin-like leakage of the phase change material was observed [55]. Moreover, the thermal properties of the samples were investigated, the thermal conductivity coefficient was 2.4561 W/m × K, thermal permeability 46.3241 kJ/kg, specific heat 2.1834 MJ/kg × K, and density 873.32 kg/m^3^. The average compressive strength from hot and cold curing was 10.3 MPa. The fabricated PCM encapsulated geopolymer composite wall showed higher soakability results compared to other conventional materials. The wall surface was slower to heat up and cool down, so the wall had lower heat loss values. Therefore, this material can be successfully used in construction sectors, it reduces the amount of consumed energy and greenhouse gas emissions [55].

**Figure 11:**
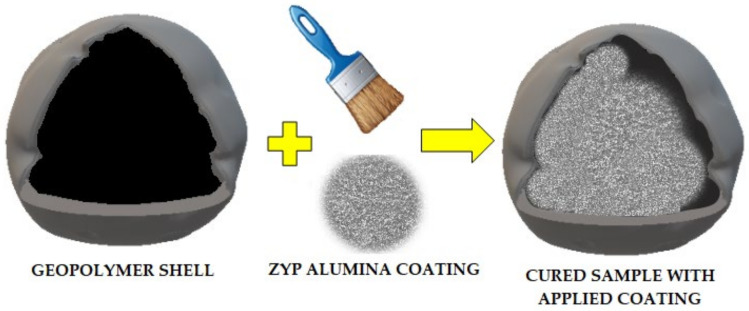
Method of applying coatings to the inside of a geopolymer semi-shell.

The effect of internal coatings on the stability of phase transformations on chloride-based materials encapsulated in geopolymers was investigated in Australia by Jacob et al., Geopolymer half-shells were produced using a mixture consisting of fly ash and black slag together with a sodium silicate-based binder. The eutectic of barium chloride, potassium chloride, and sodium chloride was selected as the phase change material. Three types of internal coatings were used: ZYP coating (Aremco Ceramacoat™ 503 VFG-C; Arecmo Products Inc., Valley Cottage, NY, USA) and ZYP YAG Binder with Merck Alumina (60G) powder (Merck, MA, USA). Hemispherical geopolymer coatings with a diameter of 25 mm and a coating thickness of 2 mm were produced and filled with 5 g of PCM. The method of coating application is presented in Figure 11 [56].

Alumina is unreactive with molten chloride-based salts, so it was anticipated that an internal coating would help reduce the loss of PCM through the coating. Weight loss of PCM through the coating was measured and a DSC test was performed on the remaining PCM to calculate a change in melting point or latent heat. Results revealed that only the alumina-coated geopolymer half shell supplied by Aremco had better performance than the control sample. This coating did not reduce PCM loss to acceptable levels (<1%). The melting point of the PCM remained mostly unchanged, but there was a significant reduction in the latent heat of all prepared samples. The Aremco coating reduced PCM loss but also caused PCM separation [56].

Kastikuas et al., introduced lightweight aggregates impregnated (LWA) with PCM for geopolymer composites made of aluminosilicate-rich silt and ground glass material. LWA expanded clay (Argex S.A.LWA) was used to produce the coated PCM-LWA. Engineered paraffin was introduced as the phase change material. PCM was introduced into the pores of LWA by the vacuum impregnation method, which was then coated with resin and granite or carbon fiber powders. Based on the results, paraffin (PCM with an approximate melting point of 25 °C) was found to be compatible for use in impregnating lightweight expanded clay aggregate with sizes of 2–10 mm. In addition, low cost and high availability were additional advantages. Vacuum impregnation and coating are simple and can be successfully performed for the desired aggregate sizes. Impregnation does not affect the melting point and solidification temperature of phase change PCM. The macroencapsulated phase change composite has a heat storage capacity similar to PCM impregnated products commonly used [19].

Aggregates that were powder-coated with resin and granite had 42% higher thermal conductivity than aggregates that were in the raw state. The improvement in thermal conductivity was not affected by modifying the resin coating with milled carbon fibers or graphite spray. The aggregates also had lower binder compressive strength. The neutral pH of the impregnated and coated aggregates indicated that they do not interfere with the strongly alkaline environment of the geopolymer binder, a highly desirable effect. Aggregates that accumulate thermal energy in microcapsules are excellent materials for wall or ceiling panels, surface cooling systems, or other structural materials [19].

Shadnia et al., investigated the effect of PCM addition on the physical, mechanical, and thermal properties of a geopolymer mortar synthesized from low-calcium fly ash (San Juan Generating Station in New Mexico), river sand (Arizona Concrete Aggregate in Tucson, AZ, USA), and microencapsulated PCM (Microtek Laboratories, Inc. in Dayton, OH, USA) at 0%, 10%, and 20% content. The results showed that the addition of PCM to the geopolymer resulted in a weight reduction, which is beneficial for the construction of lightweight buildings. Moreover, PCM addition let to a slight decrease in compressive strength, but even in the case of 20% PCM addition to geopolymer, these values were high enough for application in structural engineering. DSC analysis indicated that the introduction of PCM increased the heat capacity of geopolymer. This may contribute to the reduction of heat transport through the geopolymer mortar, and thus find application for the above material on the walls of buildings, reducing the energy demand for cooling and heating [54].

In turn, the research conducted by a group of scientists from Norway and Spain aimed to determine the effect of microencapsulated phase change materials (MPCM) as an additive for geopolymer concrete (GPC). For this purpose, MPCMs were added to a geopolymer mortar based on fly ash (Cemex, Ratingen, Germany), sand (Gunnar Holth and Skolt Pukkverk AS, Kongsvinger, Norway), ground granulated blast furnace slag (Cemex, Ratingen, Germany), and a stabilizer (FLUBE OS 39, Bozzetto Group, Filago, Italy) at different weight ratios. Three different MPCMs were compared: PS-DVB/RT27, produced by suspension polymerization (MPCM consisting of a paraffinic Rubitherm^®^RT27 core coated with PS-DVB layer) [57], PMMA/PCM26 (Micronal DS-5038X; Microtek, Dayton, OH, USA), having a core that is a mixture of paraffin and a highly cross-linked polymethyl methacrylate coating, and MF/PCM24 (Microtek MPCM24D) has a paraffin blend core and a melamine-formaldehyde (MF) polymer coating [58,59].

Other experimental results showed that with the addition of 5.2 wt.%. MPCM to GPC, the energy consumption to stabilize the internal temperature at 23 °C could be reduced by up to 18.5 ± 0.3% for GPC containing PS-DVB/RT27 (Rubitherm^®^RT27 paraffin core), 20.1 ± 0.7% for GPC containing PMMA/PCM26 (mixed paraffin core with cross-linked polymethylmethacrylate shell), and 25.1 ± 0.7% for GPC containing PMMA/PCM26. Unfortunately, the same parameters that favorably reduced energy consumption also resulted in a greater decrease in compressive strength since the core of the microcapsule were in the liquid state. However, the compressive strength still meets European standards (EN 206-1) for concrete applications (except for the sample containing 5.2 wt.% MF/PCM24) [60].

**Table 2:** Research (selected) summary of geopolymer containing PCM.

Name of PCM	Producer	Core Material of PCM	Melting Point of PCM [°C]	Heat Capacity of PCM [kJ/kg]	Form of Applied Matrix for PCM before Incorporation into Geopolymer	Application Effect in Material	References
RT31	Rubiterm, Berlin, Germany	Paraffin	31	165	Polyurethane foam, covered with geopolymer paste	Protection against overheating as a passive cooling system	[53]
MPCM 28D	Microtek Laboratories, Inc., Dayton, OH, USA	Paraffin	28	180–195	Polymer shells (microcapsules)	Damping function, less temperature fluctuation; increase in heat capacity	[54]
PCM	Avantis Company La- Boratorium Berhad, Ipoh, Perak, Malaysia	Paraffin	No data	189	Expanded graphite + CaCl_2_/sodium silicate coating (microcapsules)	Storing thermal energy and releasing it as latent heat	[55]
PCM	No data	Eutectic BaCl_2_ + KCl + NaCl	No data	215	ZYP coating or Aremco coating or ZAG coatin + geopolymer shells	Reduction in latent heat of all samples	[56]
PCM	No data	Paraffin	25	230	Commercial synthetic rubber emulsion (Sika Latex), commercial liquid waterproofing membrane (Weber dry-lastick), polyester resin adhesive with hardener and catalyst	The aggregate covered with PCM material allows for the storage of thermal energy and return it as latent hea	[19]
PS-DVB/RT27	Rubitherm, Berlin, Germany	Paraffin	24.9	100	Polymer shells (microcapsules)	Reduction in energy consumption for internal temperature stabilisation by 18.5 ± 0.3%	[60]
Micronal DS-5038X	Microtek Laboratories, Inc. Dayton, OH, USA	Paraffin	24.7	110	Polymer shells (microcapsules)	Reduction in energy consumption for internal temperature stabilisation by 20.1 ± 0.7%	[60]
RT27	Rubitherm, Berlin, Germany	Paraffin	24.9	100	Composite made by vacuum mixing hydrophobic expanded perlite (EP) and paraffin in a 1:1 ratio	Geopolymer foam concrete (GFC) showed little temperature variation and little loss	[30]

The addition of 15% and 30% PCM composite reduced the peak temperature in the test room by 1.85 °C, 3.76 °C, respectively, while increasing the heat storage capacity by 105% and 181%. Despite the reduction in mechanical properties of the geopolymer with PCM, the GFC containing PCM showed improved mechanical properties. Air bubble distribution was also improved due to the formation of uniform and fine air bubbles in the integrated GFC of PCM [30].

An important factor related to the use of phase change materials in geopolymer concrete or cement concrete is the influence of the interfacial boundary between the components. The changes of compressive strength, weight loss, and microstructure of geopolymer concrete with PCM additive related to cyclic freezing and thawing are very important. While such test results are known for geopolymer concretes and cement concretes without PCMs [61,62,63,64,65], such test results for building materials with introduced PCMs are rarely reported.

Pilehvar et al., investigated the effect of frost conditions on the physical and mechanical properties of materials modified with two types of microcapsules containing phase change materials. The authors drew attention to the fact that temperature changes under freeze-thaw conditions can cause microcracks in the transition zones, which depend on the applied microcapsule shell. The used hydrophobic materials are characterized by poor adhesion to concrete. Such a connection causes the formation of gaps, which in turn may reduce the strength properties of the material. This behavior was observed for both geopolymeric materials and Portland cement containing PCM material. During cyclic tests (freezing-thawing) in the case of Portland cement, the formation of a crystalline structure as a hydration product was observed, which decreased the strength properties of the material (this phenomenon was not observed for geopolymer material). Comparing the compressive strength of geopolymeric and Portland cement materials containing PCM material shows a decrease in strength, however, the decrease is smaller for GP, which was additionally characterized by greater stability after 28 cycles compared to Portland cement. It should also be mentioned that the introduction of phase change materials into the concrete matrix causes an increase in the porosity of the material, which may also result in a reduction in strength, as well as allowing greater water absorption [66].

Similar results confirming higher resistance of geopolymers to freeze/thaw cycles in comparison to composites based on Portland cement were presented by the authors of [67]. It was found that the weight loss was below 1% for each sample after 28 cycles of cyclic freezing. The compressive strength of PCC and GPC decreases after exposure to 28 cycles, but GPC have higher cyclic freeze strength compared to PCC. In addition, an interesting observation was made that the addition of micro-encapsulated materials (MPCM) reduces the strength loss to 2.5% for each sample after 28 days under freeze cycling, proving that MPCM offers exceptional freeze resistance. Frost-induced stresses are reduced as water expands into the free space created by air voids and pores between the microcapsules and the adjacent concrete [67].

## 5. Summary

The use of sustainable materials is unavoidable due to increasing environmental pollution and the looming ecological disaster that environmentalists have been talking about for several years now. Energy savings reduce environmental pollution and can contribute to a real impact on the environment, especially in the construction industry. The use of phase change materials described in the article is undoubtedly an interesting perspective for implementation. The addition of phase change materials to building materials can, for example, reduce their thermal conductivity by 15% which translates into significant savings in the heating and cooling of buildings [68]. In particular, in combination with geo-polymers, which have a more environmentally friendly impact than Portland cement based concretes [65], it can lead to significant improvements in performance (about 15–30% higher heat storage capacity).

Studies show that alkali-activated geopolymers have the potential to be used as thermochemical heat storage materials for both thermal energy storage sorption/desorption and hydration/dehydration. Different chemical compositions, gel structures and texture properties of geopolymers can affect energy storage performance, including charging temperature, mass/volume energy storage capacity, and charging kinetics (a critical factor for heating power) [69]. However, continuous research in this area is needed. Most authors of scientific papers describe the positive effects of PCM. There is no doubt about this. However, the problem is still the very high price of such materials. Partly this is connected with the problematic placing of this type of substance in composites. Overcoming these problems by developing different technologies has resulted in very high prices of these materials.

Currently, the use of PCM as energy storage is mainly limited to research or demonstration projects [70]. Improving the thermal comfort of buildings by adding only a few percent of PCM is more feasible in terms of implementation. However, it will still not be a widespread technology until PCM materials can be developed from cheap raw materials and at the same time in a physical form capable of being encapsulated in building materials. The challenge here lies mainly in open-pore building materials in which it is difficult to encapsulate PCM in liquid form.

Current prices for phase change materials are up to several tens of euros per kg. Of course, in the case of mass production and bulk purchases, these prices are much lower, but still at a very high level. It seems unrealistic to implement this type of material on a mass scale, mainly due to the very high costs of their use in construction. Although waxes and paraffins cost only about 2 euro per 1 kg, the purchase of processed microencapsulated materials is several or more times more expensive [71,72]. Assuming the need for phase change materials in the amount of 5% and the price of 30 euros per 1 kg, then in the case of solid concrete materials with a density of about 2300 kg/m^3^, the cost of phase change materials alone will be almost 3500 euros. Of course, in the case of foamed insulating materials this is much lower, but there are additional problems related to the way the PCM is placed inside. Another very important problem that not many researchers are yet paying attention to is the issue of PCM volume change with phase change. According to recent research results, the volume change of PCM materials can range from negligible values up to 24% [73]. This issue is not discussed in most scientific publications and reports available and it seems to be one of the most important problems that can affect the performance of the final product, because the change in volume can lead to capsule unsealing or leakage directly from the porous material. This can lead to a decrease in the amount of PCM in the building material, to an uneven distribution of this material, and to environmental contamination in case of spillage. It can also lead to damage of the microstructure of building materials and cause microcracks that reduce mechanical strength. This topic definitely needs to be addressed in detail in order to increase the real chances of PCM mass admixture in the construction industry.

Regarding the use of PCM in building materials, it can be said that the benefits of their application are very large, especially in the current period of striving to reduce CO_2_ emissions and lower the energy consumption of buildings. For many years of development PCM materials have been perfected to the extent that it is possible to use them in various areas of economy, also in construction for which a number of forms of PCM have been created, e.g., encapsulated or encapsulated, etc. However, further development connected with lowering the production costs of such materials and searching for other cheaper raw materials should be constantly pursued. In the opinion of the authors, the real application of PCM on a mass scale and noticeable to the environment will occur only in a few or several years and the key to this development will be overcoming the price barrier—reaching a level where it will be more profitable to use energy-saving but expensive materials than to bear the costs associated with CO_2_ emissions and environmental pollution.

## Figures and Tables

**Table 1:** Advantages and disadvantages of three incorporation processing routes (based on [34,49]).

	Advantages	Disadvantages
**Direct method**	No additives required, Easy, one-step process, A large amount of oil	Phase separation can take place before setting up the process
**Preemulsification**	Large quantity of oil, Controlled oil droplet size, Phase separation controlled by preemulsification	Two-step process, The addition of surfactants required
**Impregnation**	No phase separation, Leaching of toxic compounds	Two-step process, Requires addition of solid adsorbent, High cost of solid adsorbent

## Data Availability

Not applicable.
